# Impact of Endoplasmic Reticulum Stress Sensors on Pectolinarin Induced Apoptosis

**DOI:** 10.3389/fpubh.2020.00478

**Published:** 2020-09-09

**Authors:** Ji-Hye Song, Kisang Kwon, O-Yu Kwon, Eun-Ryeong Lee, Seung-Whan Kim, Kyung-Hee Kang

**Affiliations:** ^1^Departments of Anatomy and Cell Biology, College of Medicine, Chungnam National University, Daejeon, South Korea; ^2^Department of Biomedical Laboratory Science, College of Health and Welfare, Kyungwoon University, Gumi, South Korea; ^3^Department of Emergency Medicine, College of Medicine, Chungnam National University, Daejeon, South Korea; ^4^Department of Dental Hygiene, College of Medical Science, Konyang University, Daejeon, South Korea

**Keywords:** pectolinarin, PC12 cells, endoplasmic reticulum (ER), stress, sensor

## Abstract

Pectolinarin, [5,7-Dihydroxy 4′,6-dimethoxyflavone 7-rutinoside, 7-[[6-O-(6-Deoxy-α-L-mannopyranosyl)-β-D-glucopyranosyl] oxy]-5-hydroxy-6-methoxy-2-(4-ethoxyphenyl)-4H-1-benzopyran-4-one], has been stated one of the major compounds in *Cirsium nipponicum* (Maxim.) Makino. It is characterized by biological functions of hepatoprotective, anti-inflammatory and antiobesity activities. In this research, it was explained that pectolinarin causes apoptosis in PC12 cells conducted by DNA fragmentation and formation on apoptotic bodies through the activation of ER stress sensors (ATF6 fragmentation and eIF2α phosphorylation). The result of treating the PC12 cells with 50 μM pectolinarin for 24 h has come to increase ATF6 mRNA expression up to 1.6 times, PERK expression up to 1.7 times and IRE1 expression up to 1.4 times, respectively, compared to those of the control. ATF6 fragmentation by pectolinarin treatment was increased about 2 times compared with its control, and phosphorylation of eIF2α was increased 2.5 times. The results proposed that the perception of the molecular mechanisms underlying pectolinarin-caused apoptosis may be useful in new natural medicinal products and health supplements for the apoptosis-related diseases.

## Introduction

*Cirsium nipponicum* (Maxim). Makino is an asteraceae perennial herb, called Island thistle (in English) and Mul-eong-gcong-kwi (in Korean), distributed widely throughout Ul Leung Do (an island located at the east of Korean Peninsula), and it is used as a medicinal or edible plant (1). In Oriental medicine, roots are generally used for disease treatment, therefore the leaves and stems are collected when flowers are in full bloom (2). Traditionally, it has been widely used as a traditional medicinal product for the treatment of bleeding, hepatitis, hypertension and blood circulation diseases (3, 4). Recently, pharmacological studies have shown that its extract has antitumor (5) and antidiabetic (6), antioxidant (7), anti-inflammatory (8), and antifungal functions (9). This contains a significant amount of flavonoid mixtures, among which pectolinarin has been reported to be the major compound (10). The important biological activity of pectolinarin reported so far is as follows; anti-inflammatory, hepatoprotective, antiobesity activities and analgesic effect (11). However, the biological function of pectolinarin is not precisely defined.

The endoplasmic reticulum (ER) is an organelle spotted in eukaryotic cells. It is a very important manufacturing site for the post-translational step that mediates the synthesis, folding, modification and transport of secretory proteins. Some kinds of stressors that disrupt the endoplasmic reticulum function lead to accumulation of un-, mis-, misfolded proteins in the endoplasmic reticulum lumen (12). The endoplasmic reticulum stress induces ER-stress adaptable signal called the unfolded protein response (UPR) to maintain endoplasmic reticulum homeostasis *via* activation of endoplasmic reticulum chaperones, such as binding immunoglobulin protein (BiP), glucose-regulated protein 94 (GRP94), calnexin, calreticulin, endoplasmic reticulum protein 29 (ERp29), heat shock protein 47 (HSP47) and protein disulfide isomerase (PDI), which regulates three types of endoplasmic reticulum stress sensors, containing IRE1(Inositol Requiring Enzyme 1), PERK(PKR-like ER kinase) and initiating ATF6(transcription factor 6) (13–15). Though this study did not fully document the specific significance of endoplasmic reticulum stress protein expression, it demonstrated that pectolinarin controls the expression of endoplasmic reticulum stress sensors associated with apoptosis using the PC12 cells, which is widely used as a classical neuronal cell model.

## Materials and Methods

### Sample, Cell Culture, and MTT Assay

Pectolinarin (chemical formula, C_29_H_34_O_15_; molar mass, 622.57 g/mol) purified at a purity of >95.0% (HPLC) derived from *Cirsium nipponicum* (Maxim.) Makino was gifted by National Development Institute of Korean Medicine (NIKOM). The PC12 cells were cultured in collagen-coated plates or flasks containing 85% RPMI-1640 medium, augmented with 25 mM HEPES buffer, horse serum 10% heat inactivated, fetal bovine serum 5% heat inactivated, 1 mM sodium pyruvate, 1 g/l d- (+) -glucose, 2 mM L-glutamine, 25 μg/ml streptomycin and 25 U/ml penicillin (all Gibco; Thermo Fisher Scientific, Inc., USA). The cells were preserved in a humidified incubator at 37°C at 5% CO_2_ and the medium was changed every 48 h. The effects of pectolinarin on cell survival of the PC12 cells were made using an MTT kit (Sigma-Aldrich, USA). Color development was observed at 595 nm with a reference wavelength of 650 nm using the Sunrise™ microplate reader (Tecan Trading AG, Switzerland).

### RT-PCR Analysis

Each gene expression was mainly determined by RT-PCR as described below. RT-PCR conditions included 30 cycles comprising each of the following: 94°C for 30 s, 58°C for 30 s and 72°C for 1 min (10 min in the final cycle) employing the primers with *Taq* DNA polymerase (Solgent Co., Ltd., Korea). The RT-PCR primers were provided by Bioneer Corporation, Korea. The RT-PCR primers were as follows: IRE1 forward, 5′-ACC ACC AGT CCA TCG CCA TT-3′ and reverse, 5′-CCA CCC TGG ACG GAA GTT TG-3′; ATF6 forward, 5′-CTA GGC CTG GAG GCC AGG TT-3′ and reverse, 5′-ACC CTG GAG TAT GCG GGT TT-3′; PERK forward, 5′-GGT CTG GTT CCT TGG TTT CA-3′ and reverse, 5′-TTC GCT GGC TGT GTA ACT TG-3′; BiP forward, 5′-AGT GGT GGC CAC TAA TGG AG-3′ and reverse, 5′-TCT TTT GTC AGG GGT CGT TC-3′. Bcl-xl forward, 5′-CCC CAG AAG AAA CTG AAC CA-3′ and reverse, 5′-GCA GAA CTA CAC CAG CCA CA-3′; Bax forward, 5′-AGG GGC CTT TTT GTT ACA GG-3′ and reverse, 5′-GAT CAG CTC GGG CAC TTT AG-3′ Bcl2 forward, 5′-AAG CTG CAC AGC GGG GCT A-3′ and reverse, 5′-CAG ATG CCG GTT CAG GTA CT-3′ Bak1 forward, 5′-TTA CCT CCA GCA GGA AC-3′ and reverse, 5′-ACC ACC TCT CTG TGC AAT CC-3′ LC3a forward, 5′-GCC TGT CCT GGA TAA GAC CA-3′ and reverse, 5′-GTT CAC CAG GAA GG-3′ Beclin forward, 5′-GTG CTC CTG TGG AAT GGA AT-3′ and reverse, 5′-GCT GCA CAC AGT CCA GAA AA-3′ Cal forward, 5′-GGC ATC TTC ATC CCA GTC AT-3′ and reverse, 5′-CTC CTC TCT GCT CCT CAT GG-3′ PDI forward, 5′-CAG AGT TCT GCC ACC GCT TC-3′ and reverse, 5′-TCC TCG AGA TCG TCA TC-3′ ERp29 forward, 5′-CTC CTC TCT GCT CCT CAT GG-3′ and reverse, 5′-GCT CCA TGT TCA GCT TGT CA-3′ Xbp1 forward, 5′-AAA CAG AGT AGC TCA GAC TGC-3′ and reverse, 5′-TCC TCC TGG GTA GAC CTC TGG GAG-3′. All chemicals were acquired from Sigma-Aldrich; Merck KGaA. The figures show the outputs of a representative experiment in triplicate with different sampling units.

### Western Blotting

Immunoblotting was carried out according to the standard procedure. The PC12 cells treated with pectolinarin were dissolute by the addition of SDS sample buffer [62.5 mM Tris-HCl, pH 6.8, 6% (w/v) SDS, 30% glycerol, 125 mM DTT, 0.03% (w/v) bromphenol blue] and parted by SDS-PAGE. The proteins were moved to a nitrocellulose membrane, and the membrane was developed with the main antibodies. The rabbit anti-eIF2α antibody, eIF2α-P antibody and goat anti-actin antibody were acquired from Santa Cruz Biotechnology, Inc., USA. The mouse anti-ATF6 antibody was acquired from Novus Biologicals, LLC, USA. The horseradish peroxidase-conjugated anti-rabbit, anti-goat and anti-mouse IgG secondary antibodies were acquired from Santa Cruz Biotechnology, Inc. Goat anti-actin antibody was used to systematize the quantity of sample proteins. The blots were developed by utilizing an upgraded chemiluminescence Western blotting detection system kit (Amersham, Sweden). The experiments were carried out in triplicate, and the protein bands were defined employing Image J software (version 1.48; https://imagej.nih.gov/ij/).

### DNA Fragmentation Assay and Hoechst 33342 Staining

Following the treatment with pectolinarin, the PC12 cells were dissolute in 100 μl 10 mM Tris-HCl buffer (pH 7.4) having 10 mM EDTA and 0.5% Triton X-100. After centrifugation for 5 min at 16,000 x g, the supernatant dealt with RNase A and proteinase K (Promega Corporation, Madison, WI, USA). Eventually, 20 μl of 5 M NaCl and 120 μl isopropanol were put together and preserved with ice for 1 h. Following centrifugation for 15 min at 16,000 x g, the DNA pellets were dissolved in 20 μl TE buffer. The DNA samples were stuffed onto a 0.7% agarose gel and noted using a UV source after ethidium bromide (Sigma-Aldrich; Merck KGaA) staining. After treatment with pectolinarin, the PC12 cells were developed in incubator for 30 min with Hoechst 33342 (Molecular Probes; Thermo Fisher Scientific, Inc.) loading dye and washed in ice-cold 1X PBS. After following staining for 10 min, the stained cells were screened by employing a fluorescence microscope (Axio Scope A1; Zeiss GmbH, Germany) at 340 nm.

## Results and Discussion

### Cell Viability and ER Chaperone Expression

**Figure 1:**
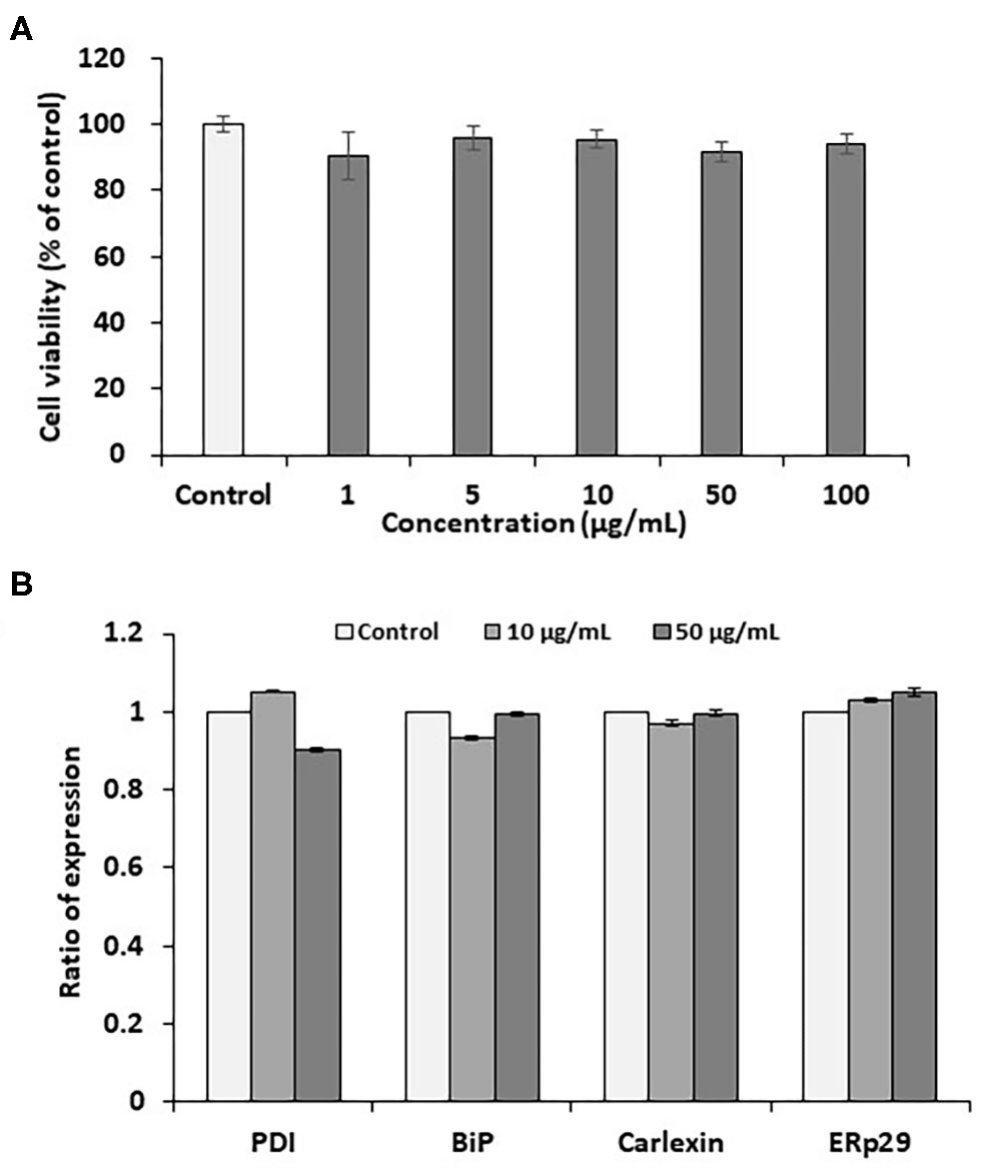
Effects of pectolinarin on cell viability and gene expression of endoplasmic reticulum chaperones in the PC12 cells. **(A)** The cells were under the incubator for 24 h with diverse concentrations of pectolinarin (0–0.1 mg/ml). Then cell viability assay was performed using MTT kit (Sigma-Aldrich). **(B)** The cells were treated with pectolinarin (10 and 50 μg/ml) for 24 h. The results of gene expression of endoplasmic reticulum chaperones of disulfide isomerase (PDI), binding immunoglobulin protein (BiP), calexin and ERp29. The RT-PCR outcomes were checked three times.

Pectolinarin is a representative component of flavonoid mixtures isolated from *Cirsium nipponicum* (Maxim.) Makino (10). It has anti-inflammatory activities and is similar in chemical structure to linarin (8, 16, 17). It is well-known that one of the remarkable features of cellular injury is leakage of soluble lactate dehydrogenase (LDH) from the cells stimulated by any stimulant. As a result of assaying, pectolinarin did not induce LDH leakage, so it was proven not to cause cellular injury within a certain concentration (18). In this study, the PC12 cell line used here is extracted from a pheochromocytoma cell of the rat adrenal medulla having a mixture of neuroblastic and eosinophilic cells. This cell line has been extensively used in *in vitro* studies to examine various neuronal diseases (19, 20). At the beginning of this study, we tested the effects of pectolinarin on cell viability in the MTT assay following at 1, 5, 10, 50, and 100 μg/ml of pectolinarin treatment for 24 h. As shown in Figure 1A, the result of the MTT assay has shown that no morphological differences were shown at concentrations below 100 μg/ml pectolinarin treatment and control cells were observed.

The endoplasmic reticulum which has newly-synthesized secretory and cell membrane proteins are post-translationally modified and correctly folded. Protein modification and folding in the endoplasmic reticulum are impaired by the endoplasmic reticulum chaperones, such as BiP, GRP94, calnexin, calreticulin, ERp29 and PDI. One of the most abundant endoplasmic reticulum chaperones in the endoplasmic reticulum lumen is BiP, a member of the Hsp70 family of proteins, which perceives newly synthesized proteins as they are transferred in the endoplasmic reticulum and sustains them in a state of competent for following folding and oligomerization. Like BiP, each endoplasmic reticulum chaperone exerts a unique function in the endoplasmic reticulum lumen to complete the correct protein folding (13–15). Although the effect of pectolinarin on cell viability was not confirmed in the MTT assay, the expression of endoplasmic reticulum chaperones (PDI, BiP, calnexin and ERp29) which indicate the degree of cell stress, was examined by pectolinarin treatment in the PC12 cells. As presented in Figure 1B, notable endoplasmic reticulum chaperone expression was not observed in the PC12 cells treated with pectolinarin. In summary, no remarkable cell viability and endoplasmic reticulum chaperone expression were observed by pectolinarin treatment on the PC 12 cells.

### Expression and Activation of Endoplasmic Reticulum Sensors

Although pectolinarin induces unremarkable cell viability and endoplasmic reticulum chaperone expression, next we have examined the gene expression of endoplasmic reticulum stress sensors (ATF6, PERK and IRE1) and the activations by pectolinarin treatment on the PC 12 cells. Under altered endoplasmic reticulum homeostasis, the endoplasmic reticulum stress signal transduction pathway is mediated *via* being active of ER(endoplasmic reticulum)- stress sensors. IRE1 activates XBP mRNA cleaving, producing an activated form of the XBP1 protein. PERK induces phosphorylation of the eIF2α, which hinders translation initiation. Active ATF6 is cleaved at the cytosolic face and the resulting N-terminal cytoplasmic domain ties to the endoplasmic reticulum stress-responding element, which enhances endoplasmic reticulum chaperone gene expression (21).

**Figure 2:**
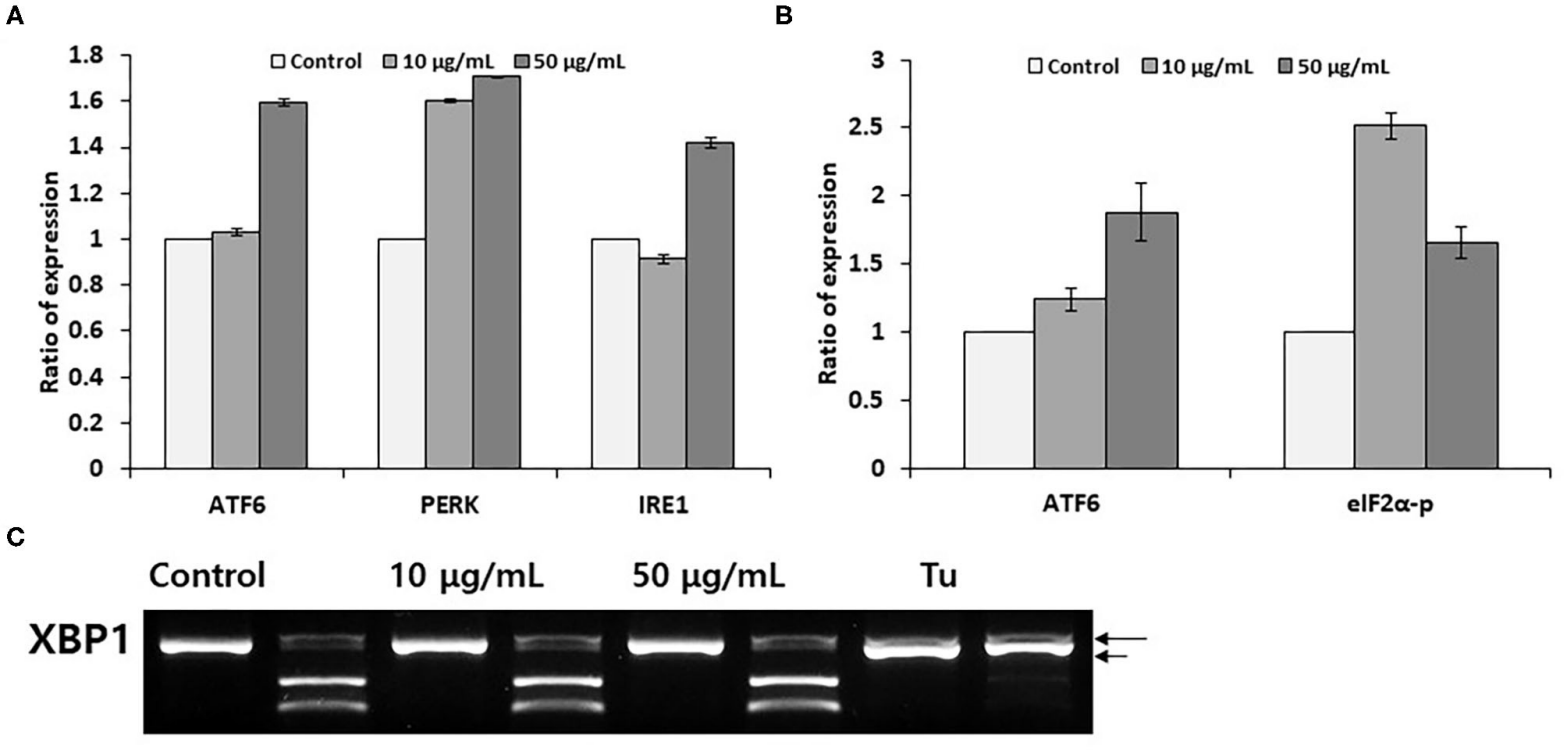
Pectolinarin commands ERSS(Endoplasmic Reticulum-Stress Sensors). **(A)** The cells were dealt with pectolinarin (10 and 50 μg/ml) for 24 h. The gene expression of endoplasmic reticulum stress sensors of ATF6, PERK and IRE1 was performed by RT-PCR. (B) Western blotting was performed using anti-eIF2-α antibody/eIF2-α-P antibody, anti-ATF6 monoclonal antibody against cells treated with pectolinarin (10 and 50 μg/ml) for 24 h, respectively. The resulting bands (fragmented ATF6 and phosphorylated eIF2-α) were estimated by Image J software (version 1.48). **(C)** RT-PCR was performed against mRNAs of XBP1. The following PCR product was moreover digested by *Pst*I to disclose a restriction site that was lost following XBP1 splicing under endoplasmic reticulum stress. The following XBP1 cDNA products were disclosed on a 2% agarose gel. Unspliced XBP1 mRNA made the two lower bands which were indicated by arrows (upper, 290 bp and lower, 183 bp). The spliced XBP1 mRNA pointed out by a bold arrow. ATF6, activating transcription factor; PERK, PKR-like ER kinase (PERK); IRE1, inositol requiring enzyme 1 (IRE1); eIF2-alpha-P, phosphorylated form of translation initiation factor eIF2α; XBP1, X-box tying protein 1, Tu, tunicamycin, respectively.

We have determined the expression of endoplasmic reticulum stress sensors under same experimental conditions as described in Figure 1B. As showed in Figure 2A, dealing the PC12 cells with 50 μM pectolinarin for 24 h increased ATF6 mRNA expression up to 1.6 times, PERK expression is 1.7 times and IRE1 expression is 1.4 times compared to those of the control, respectively. This result suggests that although pectolinarin does not regulate endoplasmic reticulum chaperone expression directly, it regulates the gene expression of endoplasmic reticulum stress sensors. ATF6 fragmentation by pectolinarin treatment was increased about 2 times compared with its control, and phosphorylation of eIF2α was increased 2.5 times (Figure 2B). However, there was a little change in the XBP1 mRNA uncleavaging that means endoplasmic reticulum stress level (Figure 2C). In summary, it has been shown that pectolinarin treatment on PC 12 cells actively regulates endoplasmic reticulum stress sensor activity through both the ATF6 fragmentation and eIF2α phosphorylation rather than the regulation of ER chaperone gene expression.

### Induction of Apoptosis

**Figure 3:**
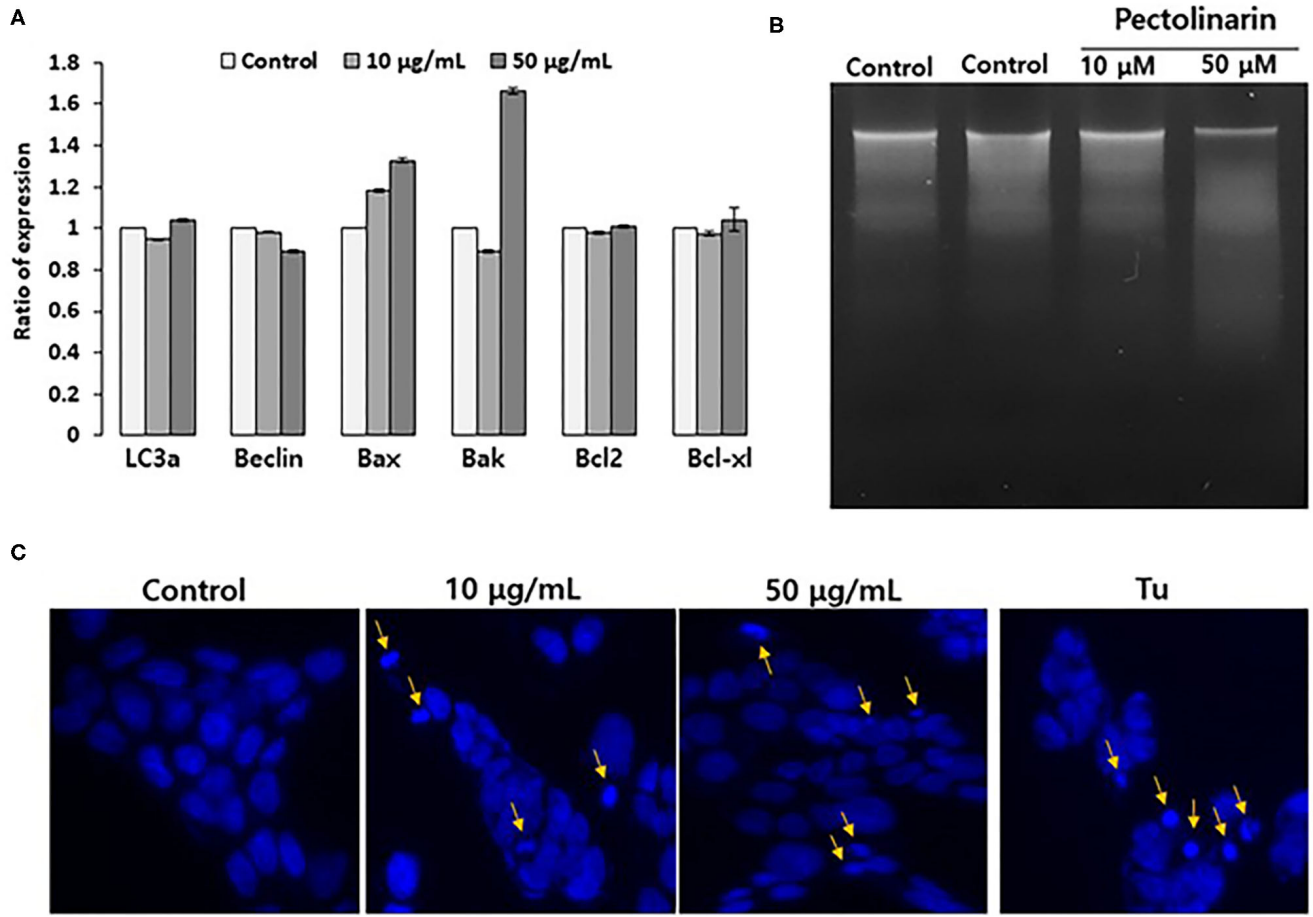
Pectolinarin causes apoptosis in the PC12 cells. **(A)** The cells were treated with pectolinarin (10 and 50 μg/ml) for 24 h. RT-PCR was performed against various mRNAs; LC3a and Beclin-1 LC3a, microtubule-associated protein light chain 3; Beclin, coiled-coil moesin-like BCL2 interacting protein; Bax, Bcl-2-associated X; Bad, Bcl-2-associated death promoter; Bcl-2, B-cell lymphoma 2; Bcl-xl, B-cell lymphoma/leukemia-x long, respectively. **(B)** To make sure DNA fragmentation, the cells were dealt with pectolinarin (10 and 50 μg/ml) for 24 h, respectively. The DNA was lysed on a 1.5% agarose gel and pictured with Ethidium bromide, an intercalating agent. **(C)** The cells were dealt with the same conditions of the above **(B)** and mixed with Hoechst 33342 solution to perceive the formation of apoptotic bodies indicated by arrows. Mixed nuclei were detected from a fluorescent microscope employing a blue filter.

If early cellular responses fail to maintain endoplasmic reticulum homeostasis, endoplasmic reticulum stress that activates UPR signal to stimulate the apoptosis as well as this autophagy for cell survival or local cell death. Already, there are already some reports that UPR strongly associated with both apoptosis and autophagy by several regulators, such as for apoptosis (Bax, Bak, Bcl2 and Bcl-xl) and autophagy (LC3a and Beclin) (22). We found that based on the results of Figure 2, nonetheless, pectolinarin induces mild endoplasmic reticulum chaperone expression, the ER stress sensors are actively induced. These findings provide new insights that the mild endoplasmic reticulum stress through PERK-eIF2α-p or/and ATF6 fragmentation indicating pathway has a main role in saving cellular damage from pectolinarin. We therefore tested the role of instantaneous endoplasmic reticulum stress in both apoptosis and autophagy induction of bystander cells treated with pectolinarin. Figure 3A showed that the pectolinarin induces only the expression of pro-apoptosis (Bax and Bak), no meaningful expression of anti-apoptosis (Bcl2 and Bcl-xl) and autophagy (LC3a and Beclin) induction. The result may provide an evidence indicating that pectolinarin induces apoptosis through endoplasmic reticulum stress signaling but not autophagy.

Apoptosis shows two typical cell changes, it morphologically makes apoptotic bodies by cell shrinkage and chromosomal DNA fragmentation. It is being used as a useful marker for identification of apoptotic cells detection of both apoptotic bodies by microscope and apoptotic DNA fragmentation *via* the DNA laddering assay (23). In this study, to confirm that pectolinarin induces apoptosis, we investigated PC12 cells treated by pectolinarin that shows apoptotic bodies and DNA fragmentation or not. As a result, inter-nucleosomal DNA fragmentation increased in cells treated with pectolinarin dose-dependently (Figure 3B). Moreover, apoptotic bodies were observed following Hoechst 33342 staining (Figure 3C). The above results clearly revealed that pectolinarin participates in the induction of PC12 cells apoptosis.

In conclusion, pectolinarin dealings obviously caused apoptosis through the expression of ERSS(Endoplasmic Reticulum-Stress Sensors) in the PC12 cells. However, at this time, it is difficult to adequately explain the signaling through endoplasmic reticulum stress triggering several regulators connected with the apoptosis pathway for the extended endoplasmic reticulum stress. It is thought that better understanding of the biological mechanisms focusing pectolinarin-caused apoptosis may be helpful and useful in the diagnosis and treatment of apoptosis-related diseases based on new natural medicinal products.

## Data Availability Statement

The datasets presented in this study can be found in online repositories. The names of the repository/repositories and accession number(s) can be found in the article/supplementary material.

## Author Contributions

J-HS gave the conceptual idea. KK developed the methodology work. O-YK helped in implementation work. E-RL helped in result analysis work. S-WK helped in documentation work. K-HK helped in final editing and drafting of manuscript. All authors contributed to the article and approved the submitted version.

## Conflict of Interest

The authors declare that the research was conducted in the absence of any commercial or financial relationships that could be construed as a potential conflict of interest.
